# A Cross University-Led COVID-19 Rapid-Response Effort: Design, Build, and Distribute Drexel AJFlex Face Shields

**DOI:** 10.1007/s10439-021-02743-w

**Published:** 2021-02-26

**Authors:** Amy L. Throckmorton, Ellen J. Bass, Bryan Ferrick, Arun Ramakrishnan, Scott Eichmann, Nicholas Catucci, Bradley Eshelman, John McNamara, Erik Sundquist, Nathan Beatson, Matthew Hirschhorn, Pooja Menon, Elizabeth Datner, Randy Stevens, Michele Marcolongo

**Affiliations:** Drexel University, Philadelphia, PA USA

**Keywords:** Personal protective equipment, PPE, Medical face shields, COVID-19, Social entrepreneurship, Shields, Design-build, Micro-supply chains, 3D printing, Participatory design

## Abstract

The purpose of this article is to demonstrate how a new cross-community leadership team came together, collaborated, coordinated across academic units with external community partners, and executed a joint mission to address the unmet clinical need for medical face shields during these unprecedented times. Key aspects of this success include the ability to forge and leverage new opportunities, overcome challenges, adapt to changing constraints, and serve the significant need across the Philadelphia region and healthcare systems. We teamed to design-build durable face shields (*AJFlex Shields*). This was accomplished by high-volume manufacturing via injection molding and by 3-D printing the key headband component that supports the protective shield. Partnering with industry collaborators and civic-minded community allies proved to be essential to bolster production and deliver approximately 33,000 face shields to more than 100 organizations in the region. Our interdisciplinary team of engineers, clinicians, product designers, manufacturers, distributors, and dedicated volunteers is committed to continuing the design-build effort and providing Drexel *AJFlex Shields* to our communities.

## Background and Motivation

During the early Spring of 2020, the immediate impact of the COVID-19 pandemic led to a startling global shortage of personal protective equipment (PPE) for healthcare workers. At the outset of the pandemic, the transmission mode, whether airborne or through contact, was unclear. Headlines across the country painted a dire picture of the ongoing struggle of healthcare providers to secure proper PPE: protective face shields, face masks, N95 respirators, ventilators, and other critically important medical equipment.^15^, ^4^, ^16^, ^3^ Global and domestic PPE supply chains were stressed because of rising demand, panic buying, and restricted delivery from mass-manufacturing countries like China, producer of approximately half of the world’s face masks.^15^,^16^ An article in the *New England Journal of Medicine* chronicled the obstacles faced by a physician executive attempting to secure a full shipment of N95 masks, only to receive 25% of the original order.^2^ EMS personnel, nurses, physicians, and other healthcare providers were being asked to reuse their single-use face masks, face shields, and respirators, thus compromising their safety. The high incidence of healthcare personnel becoming infected compounded the crisis, as it created a critical shortage of medical professionals. This situation was further complicated by additional PPE needs from organizations trying to reopen under the safest possible conditions, such as shelters, grocery stores, social establishments, and schools.

Facing these crippling challenges and dwindling inventories, grassroots rapid-response efforts to address the shortfall in our domestic production of PPE began to emerge all over the country. Colleges, universities, companies, and individuals formed new interdisciplinary teams that were inspired and mission-driven to design and build PPE for healthcare workers in desperate need of protection against COVID-19.^10^, ^9^. 6, 17 Companies like Nike, Ford, and Apple joined this sweeping movement.^10^ Many of these organizations focused their collective and collaborative efforts on the production of face shields, utilizing 3D printing technology to print headbands and assembling other key components to construct a complete face shield.^9^,^13^

Face shields protect mucous membranes and are proven to extend the lifetime of other PPE, such as face masks and N95 respirators. This type of PPE significantly limits the inhalation exposure of the wearer and reduces the surface contamination of an N95 respirator worn in conjunction with the face shield. When a person wears a face shield, inhalation exposure to large, viral-laden aerosols is reduced by 96%, and surface contamination of an N95 mask reduces by 97%.^12^ Therefore, face shields not only can protect healthcare workers but also can assist in the overall containment of COVID-19 within a community because of their efficacy and because they are relatively easy to manufacture.^14^

In the Philadelphia region, Drexel University’s *AJFlex Face shields* project quickly emerged in late March, led by Dr. Michele Marcolongo of Materials Science and Engineering in the College of Engineering, Dr. Amy Throckmorton of the School of Biomedical Engineering and Health Systems, and Dr. Ellen Bass of the College of Nursing and Health Professions and the College of Computing and Informatics. This cross-institutional leadership team rapidly mobilized and leveraged their design ingenuity, engineering knowledge, community partnerships, and technical resources to successfully design, build, and distribute tens of thousands of face shields. The purpose of this article is to demonstrate how this cross-community, leadership team came together, collaborated, adaptively coordinated across Drexel units and with external community partners, and executed a joint mission to address this unmet clinical need for face shields during these unprecedented times. Key aspects of this success include the ability to create and leverage opportunities, overcome challenges, adapt to changing constraints, and serve the significant need across the Philadelphia region and healthcare systems. This all began as a response to a clinician’s plea.

## A Clinician’s Plea: Clinical Need

On March 24, 2020, Dr. Elizabeth Datner, Chair of the Department of Emergency Medicine at Einstein Healthcare Network in Philadelphia, contacted Dr. Marcolongo. Her department was facing a critical shortage of face shields as the supply chain was severely leveraged, and she needed immediate assistance. Within 48 hours, Dr. Marcolongo had teamed with Dr. Throckmorton, Dr. Bass, and Bryan Ferrick, a recent graduate of Biomedical Engineering program at Drexel. Dr. Marcolongo and Dr. Throckmorton focused on the design through iterative prototyping and identification of materials and supplies; Mr. Ferrick kickstarted the 3D printing effort to produce the headband components (structural support for the face shield); and Dr. Bass addressed communication through the project website and listserv creation. This divide-and-conquer approach leveraged individual strengths. Using the initial design, the team delivered their first batch of 90 *AJFlex Face shields* to Einstein Healthcare Network, Penn Medicine, and St. Christopher’s Hospital for Children on April 3, 2020. This rapid response and effective teaming led to the beginning of a cross-campus effort that culminated in the donation of thousands of face shields and in the building of new community partnerships across the region. This, in turn, inspired others to contact the team with requests for Drexel face shields.

## Challenges

We originally conceptualized the design of the face shield to have five components: (1) structural supportive headband to maintain distance between the shield and the wearer’s face; (2) a layer of foam tape on the inner surface of the headband to provide forehead comfort; (3) a soft elastic band that wrapped around the back to the head and connected to both ends of the headband for head size variance; (4) cords or cable ties to connect the elastic banding to either side of the 3D printed headbands for ease of manufacture and durability; and (5) the plastic shield itself. We set an initial production goal of 6000 face shields. However, in late March and early April, we faced several immediate challenges along our roadmap to success:

1. *Shutdown and Supply Chain Constraints*: State government agencies were pivoting to fully shut down businesses and states began to issue stay-at-home orders, resulting in supply chain constraints that made it more difficult to obtain the materials to build these face shields. At the same time, we recognized that other grassroots efforts across the country were also building shields for their regional hospital systems. This situation led to the limited availability of raw materials across the board.
2. *3D Printers at Drexel and the surrounding area*: Mr. Ferrick was making headbands with 3D printers at his house, and he recruited two other 3D printing hobbyists and neighbors (Kim Jacquay and Paul McMaster). We recognized that to scale production, we would need to engage additional headband printing teams on Drexel’s campus and in the Philadelphia region and Delaware Valley.
3. *Marketing and Outreach*: Early in the effort, we also realized that what was happening at Einstein was the tip of the iceberg for our region. On the demand side, teaming with clinicians and healthcare organizations would be essential to understanding and meeting the clinical needs. On the supply side, forming community partnerships and teaming with other local grassroots efforts were also critical. This included engaging our broad professional networks to secure materials for the face shields. To expedite communications, we prioritized the design and launch of a website. This allowed us to share our mission externally and to engage volunteers, donors, 3D printing hobbyists, and organizations in need in the Philadelphia region and Delaware Valley.
4. *Design-Build-Assembly Space*: To achieve our distribution and production level while heeding social distancing precautions, we required a large warehouse space for material acquisition, manufacturing, assembly, inventory, and distribution.
5. *Scaling Production*: While our smaller-scale production was impactful, we anticipated that the establishment of corporate manufacturing partnerships would be critical to rapidly scale our production using high-volume manufacturing techniques, such as injection molding.

## Opportunities

Drs. Throckmorton, Marcolongo, and Bass formed a team under a shared mission to build and donate face shields in rapid response to the pandemic: Dr. Marcolongo led outreach, networking, and marketing; Dr. Throckmorton concentrated on the design, production, assembly, and distribution of the face shields; and Dr. Bass led the media, website design, and information technology aspects of the project. Through frequent communication, collaboration, and adaptive problem solving, the three leaders developed strategy and executed their goal-oriented approach.

**Figure 1:**
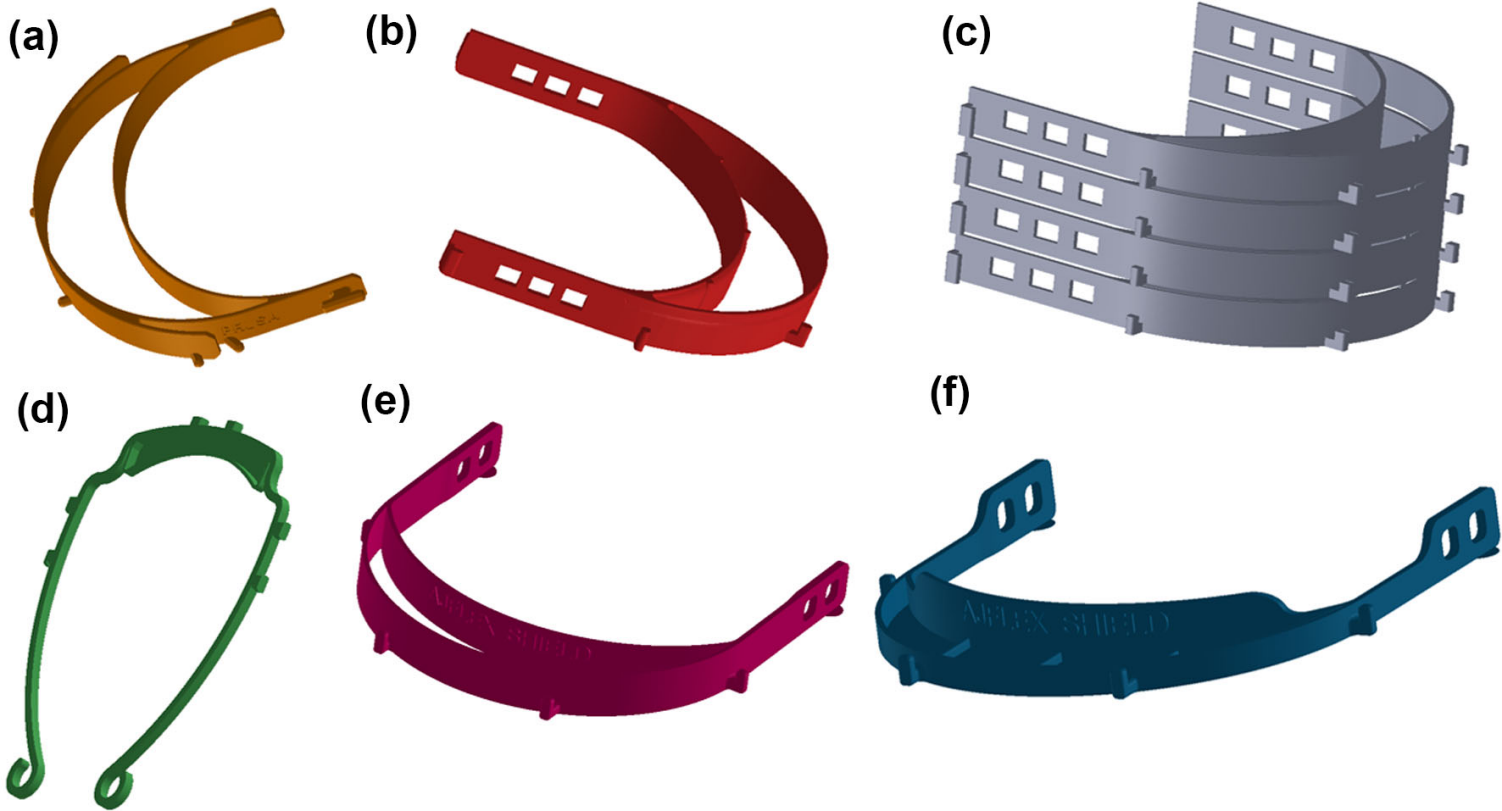
Design Evolution of the Headband for the *AJFlex Face shields*: (**a**) Prusa RC2 open-source model; (**b**) European open-source headband design with stronger support strut hooks; (**c**) Stacking example of the European headband to maximize 3D printing and production; (**d**) Verkstan headband design with stacking capabilities of 20 on a reasonably sized 3D print bed; (**e**) Modifications to the wall and internal thickness of the European headband design, as well as the hole position and number; (**f**) Drexel’s headband design with thickness optimization for increased printing speed, hole number reduction, support strut hooks, and a filled cap-like structure to further protect from airborne aerosol particulates.

Since COVID-19 cases were surging in Europe before the United States, healthcare organizations were ahead of us in dealing with PPE shortages. Dr. Marcolongo and Dr. Datner identified an open-source headband design (Fig. 1) from Prusa Research, a Czech organization. In consideration of shield material and connectors of banding, our team explored the use of double-layered lamination materials from FedEx for the shield and the incorporation of hooks and clips for the final assembly.^7^ Business shutdowns were commencing, and we knew that local hardware stores could be helpful to source materials and supplies.

Drexel University was also closing and moving its operations, teaching, and learning to the virtual and remote space, and Dr. Throckmorton needed access to 3D printers in order to generate headband prototypes. Since the United States was behind Europe and Asia in the initial wave of the virus, Drs. Throckmorton and Marcolongo found more headband designs online that could be used (Fig. 1). Dr. Throckmorton engaged Mr. Ferrick and his team to print several of these headband designs and to kickstart prototyping.

While design concepts were being considered and prototyping was beginning to quickly unfold, local news outlets took notice of Drexel University’s efforts to alleviate supply chain stress brought on by the pandemic. Dr. Marcolongo appeared on both CBS3 and 6ABC to describe our ongoing efforts and to ask for community assistance. Through these local news outlets, the team promoted their efforts and engaged numerous volunteers, healthcare workers, industry partners, and the Maker community (encompassing 3D printing hobbyists).^9^,^5^,^18^ Recruitment of these individuals and organizations facilitated rapid scaling of printing, production, and prototyping such that more healthcare institutions could be better served.

With media publicity, there was a flurry of emails, texts, phone calls, and related communication. For example, we received requests for shields from multiple healthcare organizations, and we needed to coordinate production and touchless delivery. The volunteer 3D print hobbyists sought CAD files for acceptable headband designs and needed to deliver these printed bands safely and inexpensively to our production line. University leaders and communication teams requested up-to-date information about the number of face shields that were being distributed. University employees, companies, and regional citizens sought to make financial and material donations. Thus, with her College of Computing and Informatics colleagues, Dr. Bass designed and implemented a project website to address the wide range of stakeholder needs. After conducting interviews with key user representatives, Dr. Bass delivered functional, database, and human-computer interaction requirements to the College of Computing and Informatics’ team (John McNamara, Joe Adair, Kristen Glaser, and John Sinclair). After a short iterative development and test cycle, the project website’s key functionality was deployed.^8^ Dr. Throckmorton suggested adding a way to include other media (photographs and links to news clips) and a method to honor our “Healthcare Heroes,” These new functions were added the following week. In addition, a Drexel *AJFlex Face shields* listserv was created for email.

Dozens of printing team members were 3D printing hobbyists located all over the region. However, with mounting pressure to scale their printing output, their filament inventory was quickly depleted. Thus, Dr. Marcolongo forged a corporate connection with Matterhackers, a California-based company with a passion for 3D printing. Matterhackers provided access to high-quality filament for our 3D printing hobbyists.^1^

The team pursued unconventional avenues for acquiring other scarce source materials. Community support and partners were vital to the success of Drexel’s *AJFlex Shield* project, and collaborators across the region worked together to solve the supply chain issues. For example, a Drexel team member’s relative with ties to the garment industry helped with the acquisition of the elastic banding material. A Facebook friend at a New Jersey bulk plastics distribution company provided access to a range of plastic shield materials to meet various clinical needs. Temple University generously donated two large, industrial-sized rolls of polycarbonate material, enough for approximately 2400 shields.

Other immediate actions included identifying shield materials through large distribution companies and plastic supply companies. Initial orders for polycarbonate shield material were placed through McMaster-Carr and using Amazon. The use of local suppliers to source key materials became critically important to our success because of the scarcity of resources and unknown lead times on pending orders. Dr. Throckmorton had established relationships with managers at New Jersey locations of The Home Depot and Lowe’s for materials and supplies. For production of full face shield prototypes, local Lowe’s and Home Depot stores donated thousands of dollars in door frame weather-seal soft foam strips, glue, and electrical cable ties.

Injection-molding offered a high-volume manufacturing capability to produce headbands. Subsequent batches of face shields leveraged new partnerships, forged by Dr. Marcolongo, with high-volume manufacturing companies (Southco and Engineering Plastics Corporation) in the region. These companies produced headbands using injection molding techniques and provided thousands of headbands to Drexel. These corporate partners were key contributors to the team’s boosting of production.

**Figure 2:**
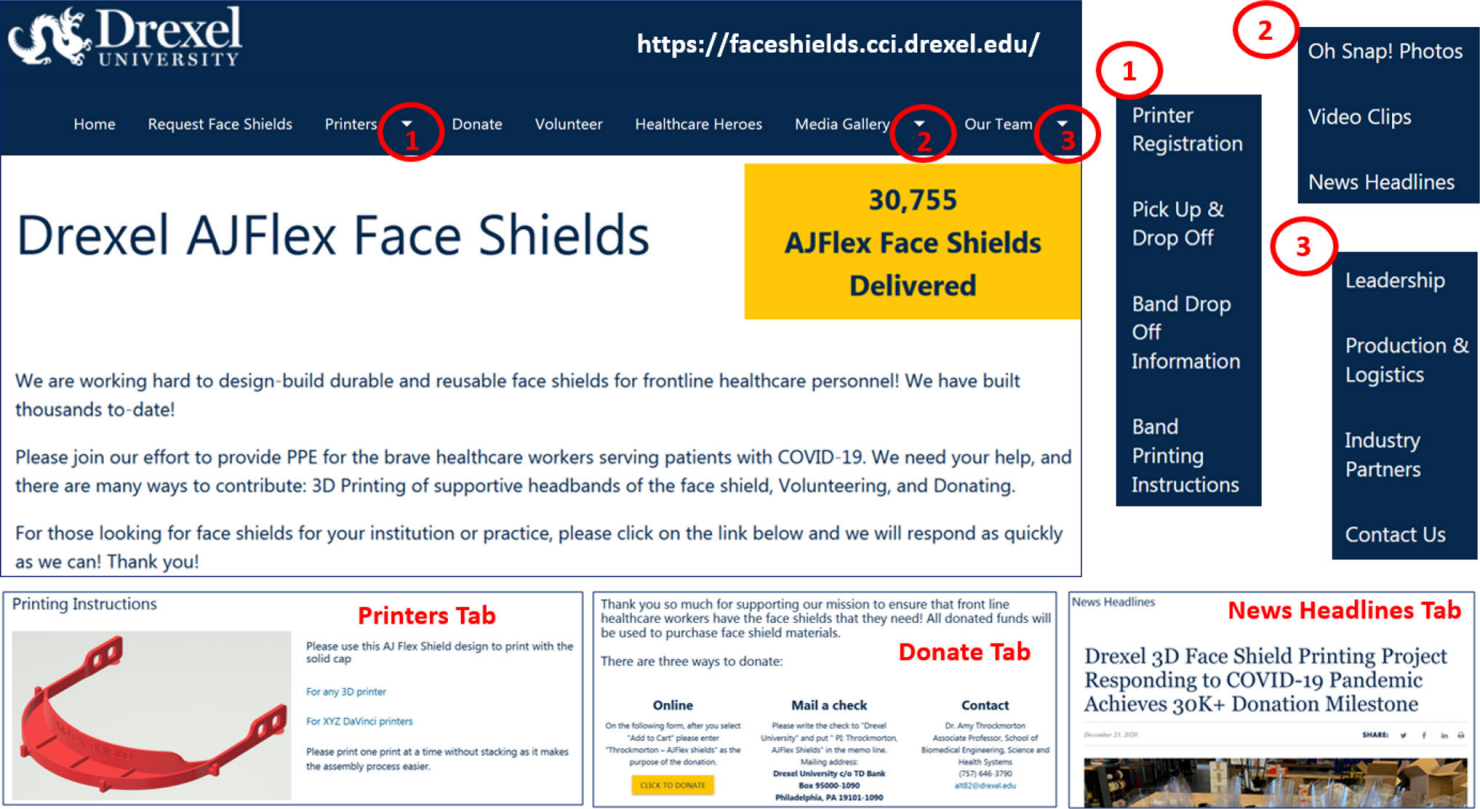
Face shield project website design and structure. Image depiction of an example of sections and sub-sections of the project website.

In only a few days, we were able to 3D print headbands and build and assemble our first batch of durable *AJFlex Face shields* (Fig. 2). While Dr. Throckmorton and Dr. Bass assembled the first shields at their homes, essential personnel designations were approved and made it possible to expand to the Maker Space in the Innovation Studio at Drexel University. This enabled the cross-campus team effort to ramp production in a staged assembly process that maintained COVID-19 physical and social distancing and University restrictions and requirements. The warehouse size of the Innovation Studio permitted us to arrange multiple production stages of assembly for the face shields, from deburring 3D printed headbands to connecting the elastic bands to adding a comfort foam strip to mounting the shield material to placing the FDA-labelling tag. Faculty, staff, and students from the School of Biomedical Engineering, Science and Health Systems; College of Nursing and Health Professions; College of Engineering; College of Medicine; Westphal College of Media Arts and Design; Office of Research and Innovation; and the Drexel Machine Shop worked together to build *AJFlex Face shields* in the Innovation Studio. As production ramped, the proximity of the production line to the Drexel Machine Shop became invaluable. Nick Catucci and Scott Eichmann, Master machinists, worked to build design-specific tools in order to ramp production time. These tools included: 1) a rapid hole-punching press to ensure successful and quick mounting of the shield materials to the headbands, and 2) a unique cutting blade set to puncture holes in the elastic bands for mounting to the headbands; this was accomplished using spare materials and parts that were readily available in the shop and using their design ingenuity.

## Design-Thinking and Adaptations

**Figure 3:**
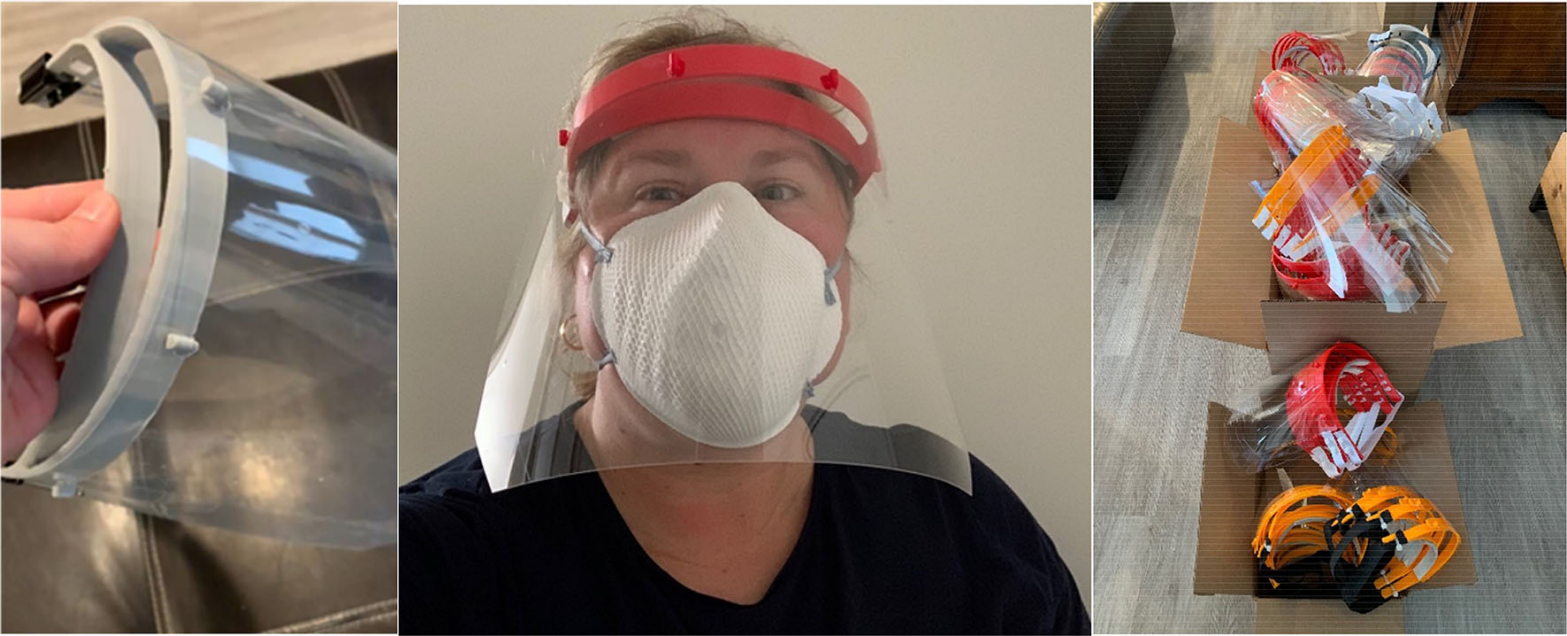
Drexel *AJFlex Face shield* with parts / components: headband, shield material, elastic banding around the back of the head, zip ties to secure the elastic material to the headband, and thin foam strip on the inner surface of the headband for comfort along the forehead.

Dr. Throckmorton gathered design requirements for a protective face shield and worked closely with Dr. Marcolongo to realize the design attributes for clinicians. The face shield headbands were produced by two techniques: (1) 3-D printing techniques (initially and ongoing), including fused deposition modeling or fused filament fabrication; and (2) high-volume manufacturing via injection molding (later due to mold design and ongoing) (Fig. 3). Plastic protective shield made from polyethylene terephthalate or polycarbonate (0.015” or 0.02” thickness) is attached to the headband using strut supports. Elastic banding, secured using electrical zip tie connectors, on the distal ends of the headband holds it in place for the user. To add a layer of comfort, we placed foam tape along the inner surface of the headband that would be in contact with the forehead. To support different head sizes and to mitigate supply chain challenges, the options for elastic banding, foam tape, and zip tie connector components are adapted. The majority of competing face shield designs are intended for single use. This design can be reused by a single wearer after disinfecting, which can be achieved with a variety of available cleaners. Moreover, the headband and strut supports for the shield are robust, allowing for head motion and for retardation of damage due to daily wear and tear.

In parallel with high-volume injection molding, cross-campus and regional print teams continued to 3D print headbands, and Drexel iterated designs of the headband after we received feedback from clinical end-users. Figure 1 illustrates the design evolution of the headband support for the shields. Initially, we leveraged readily available open-source headband designs that we found online from sources in Europe. The Prusa RC2 headband model (Fig. 1a) was used to kickstart our printing and production of headbands; this design was robust and the strut prongs were aligned with a commercially available 3-hole puncher for mounting the shield material. These strut prongs, however, became a weak point of the design, since the shield itself could easily dislodge if bumped or contacted. Therefore, we found and utilized another European open-source headband design (Fig. 1b) that incorporated much stronger support strut hooks, rather than prongs. We had also developed a method for stacking these European headbands to maximize 3D printing and production (Fig. 1c). At the same time, the Verkstan headband design (Fig. 1d), a thinner and less complex shape, surfaced, and we tried several of these, realizing that our 3D print beds could accommodate a print stack that was 20 deep. After testing these Verkstan headbands, we received feedback that (1) the shield fogged easily, since the shield is closer to the face; (2) end-users developed tension headaches more frequently; and (3) the use of support prongs rather than hooks in this design limited long-term shift usage, since the shield could more easily be bumped from the headband. Additionally, because of a growing global demand for 3D printing projects, there was a severe shortage of 3D printers and filaments spools (e.g. ABS and PLA).

Dr. Ramakrishnan collaborated with Dr. Throckmorton and Dr. Bass to develop our own Drexel headband design (Fig. 1e) by integrating the positive design attributes of all of these headbands. He was able to quickly thin the wall thickness and internal thickness characteristics of the European headband design with the strut hooks. He reduced the hole number and position for the elastic bands, removed the last set of connector struts near the holes, and reduced the overall print filament requirement by 30%. He evaluated different print speeds and temperatures to improve print time, and he incorporated a filled cap-like or shade structure to further protect from airborne aerosol penetration (Fig. 1f). He also installed additional 3D printers and developed a printing guide with optimal parameters for fast and repeatable prints, which was posted on the website. In addition, we included a logo on our final print design.

## Community and Healthcare Impact

**Figure 4:**
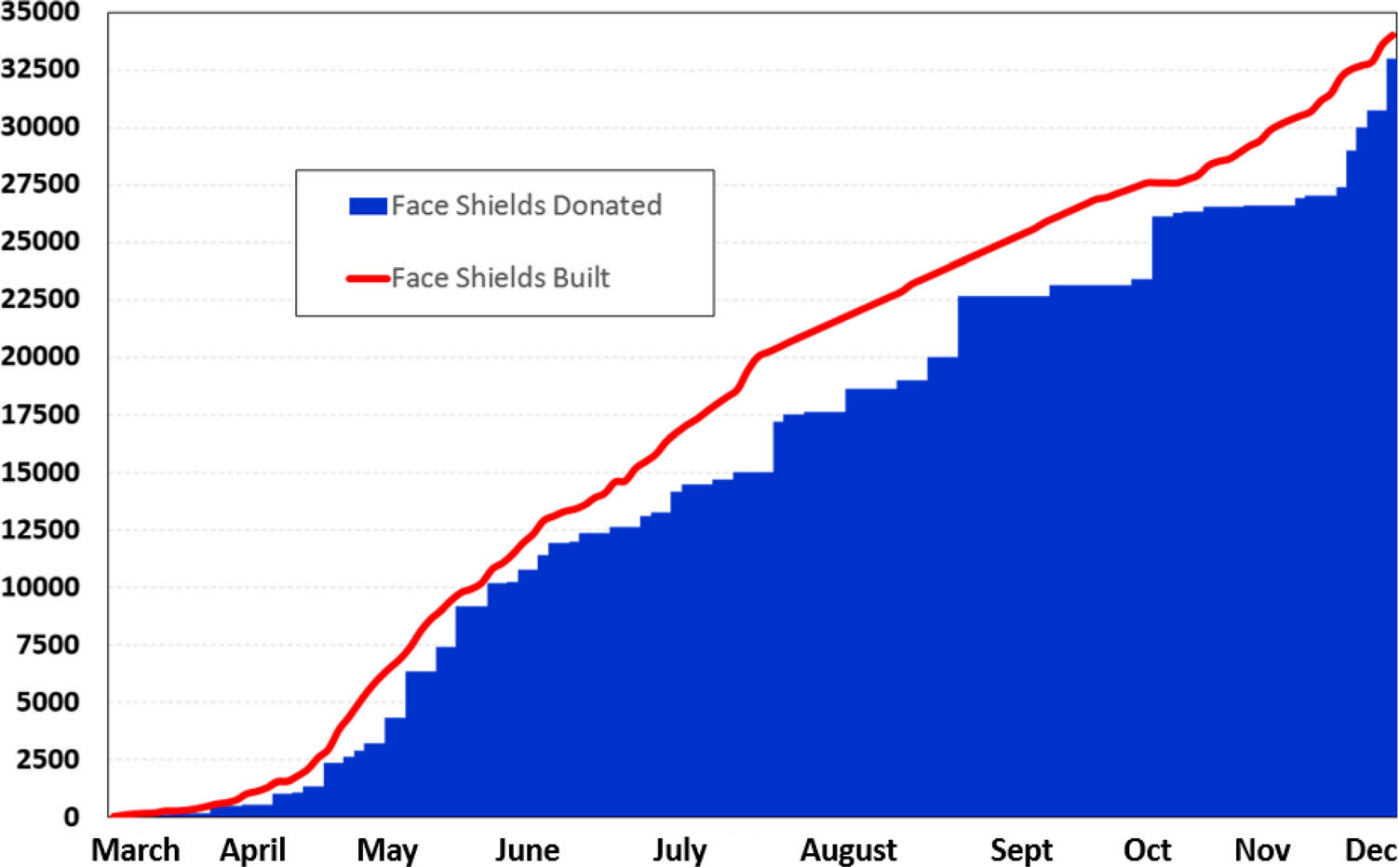
2020 Distribution of the Production and Assembly of *AJ Flex Face shields* and the Distribution of Shields to Regional Organizations Since the Project Started. We have built 34,000 and donated 33,000 shields, respectively.

In collaboration with industry partners, this cross-campus and community-wide effort has resulted in the production of approximately 34,000 Drexel *AJFlex Face shields* and delivery of 33,000 face shields to more than 100 organizations. These organizations include EMS/police departments, hospital systems, nursing and rehabilitation facilities, educational institutions, and shelters and community non-profit organizations. Weekly face shield requests from these organizations have varied from 500 to 3000 per week since the first week of April 2020. Figure 4 illustrates our face shield design, manufacturing, and production success to date.

Table 1 lists locations that received our face shields. To highlight, Virtua Health Systems at Our Lady of Lourdes and Willingboro received 2000 of our face shields in order to provide added protections to staff and visitors. We supplied a Cooperative Educational Program that supports the education and professional development of at-risk teens with 1500 face shields. The Corp. Michael J. Crescenz VA Medical Center in Philadelphia received 1,000 face shields as part of an effort to stockpile reserves of PPE in preparation for this coming winter season. By October 2020, we had distributed approximately 3500 shields to the Philadelphia School System as it prepares plans for a return to the classroom.

**Table 1:** List of hospitals, rehabilitation and healthcare facilities, educational organizations, and non-profit institutions that received donations of the *AJ Flex Face shields*.

AIM Academy, Philadelphia, PA	Immaculate Mary Center for Rehab. and Healthcare, Philadelphia, PA
API 3101 Building Personnel, Philadelphia, PA	Jenks Academy for Arts & Sciences, Philadelphia, PA
Aventura Nursing Facility, Prospect Park, PA	Lankenau Hospital Neonatal Intensive Care Unit, Wynnewood, PA
Barclays Health Services, Cherry Hill, NJ	Lutheran Community at Telford, Telford, PA
Bassett Medical Center, Cooperstown, NY	Manheim Township Schools, Lancaster, PA
Bethesda Philly Combat Homelessness Project, Philadelphia, PA	Manorcare Health Services, Voorhees, NJ
British International School in Ethiopia	Medford Leas Senior Living, Medford, NJ
Camden County, Police Department, Camden, NJ	Oak Street Health, Philadelphia, PA
Cherry Hill Nursing Home Facility, Cherry Hill, NJ	Overbrook Educational Center, Philadelphia, PA
Children’s Hospital of Philadelphia, Philadelphia, PA	Philadelphia Sexual Assault Center, Philadelphia, PA
Christiana Hospital System, Wilmington, DE	PA Department of Health, Harrisburg, PA
Cigna Testing Group, Cherry Hill, NJ	PA Emergency Management Agency, Harrisburg, PA
Crescenz VA Medical Center, Philadelphia, PA	Paulino Home Healthcare, Philadelphia, PA
Crossroads School Hunting Park, Philadelphia, PA	Penn Medicine, Philadelphia, PA
Eastside Medical Center, Erie, PA	Penn Medicine COVID Care Force, Philadelphia, PA
Einstein Health System, Philadelphia, PA	Radford Carilion Hospital Rehabilitation, Radford, VA
Elmwood Hills Healthcare Center, Blackwood, NJ	Right-at-Home Healthcare Services, Cherry Hill, NJ
Evergreen Elementary, Woodbury, NJ	Samost Jewish Family & Children’s Service, Cherry Hill, NJ
Delaware Premier Pediatrics, Newark, DE	Silver Healthcare Center, Cherry Hill, NJ
Drexel Autism Institute, Philadelphia, PA	Southeastern Cooperative Educational Programs, Virginia Beach, VA
Drexel Medicine Caring Together Program, Philadelphia, PA	Temple University Emergency Department, Philadelphia, PA
Drexel University College of Medicine, Philadelphia, PA	Temple University Hospital – Endocrinology, Philadelphia, PA
Drexel University College of Nursing and Health Professions, Philadelphia, PA	The Pines at Philadelphia - Rehab and Healthcare Center, Philadelphia, PA
Drexel University Laboratory Animal Resources, Philadelphia, PA	Total Care Physicians, Newark, DE
Drexel University LeBow College of Business, Philadelphia, PA	St. Christopher’s Hospital for Children, Philadelphia, PA
Drexel University Police Department, Philadelphia, PA	St. Mary’s Center for Rehabilitation and Healthcare, Cherry Hill, NJ
Drexel University Residential Team, Philadelphia, PA	Unitarian Universalist House Outreach Program, Philadelphia, PA
General Surgery, COVID Unit, Einstein Hospital, Philadelphia, PA	Virtua Our Lady of Lourdes Hospital, Camden, NJ
Hammonton Center for Rehabilitation, Hammonton, NJ	Virtua Willingboro Hospital, Willingboro, NJ
Holy Redeemer Hospital, Meadowbrook, PA	White Horse Village, Newtown Square, PA

### Conclusions

Rising to the challenge of broken PPE supply chains in unprecedented times, faculty leaders and collaborators at Drexel University and in the local community joined together to deliver 33,000 face shields. Drexel’s *AJFlex Shields* were made using 3D printing and injection-molding techniques and assembled with polycarbonate or PET plastic sheeting. They were intended to be used as a stop-gap measure until supply chains were restored. Partnering with industry collaborators and community partners proved to be essential in bolstering production and providing for over 100 organizations in the region. While these efforts have focused on protecting healthcare workers, future endeavors can look to moving face shields into the community as a potential alternative to masks. The 3D printing and injection molding headband components were found to work and were integrated into the face shield design to provide robust support. Rapid feedback about the designs allowed for improvement in function and usability.

Our work is not finished. Hospitals and rehabilitation facilities are currently experiencing a higher volume of patients due to increased transmission and infection rates. These facilities are preparing for the resurgence that we are witnessing now and for additional waves to occur in the Winter season until the vaccines are widely available and distributed. Our interdisciplinary team of engineers, clinicians, product designers, manufacturers, distributors, and dedicated volunteers is committed to continuing the design-build effort of the *AJFlex Shields* for our regional communities. In future pandemics or catastrophic events, Drexel University and partners offer hope to the community it serves.
